# Designing a Microfluidic Device with Integrated Ratiometric Oxygen Sensors for the Long-Term Control and Monitoring of Chronic and Cyclic Hypoxia

**DOI:** 10.3390/s150820030

**Published:** 2015-08-14

**Authors:** Samantha M. Grist, Jonathan C. Schmok, Meng-Chi (Andy) Liu, Lukas Chrostowski, Karen C. Cheung

**Affiliations:** Department of Electrical and Computer Engineering, The University of British Columbia, 2332 Main Mall, Vancouver V6T 1Z4, BC, Canada; E-Mails: sgrist@ece.ubc.ca (S.M.G.); jonathan.schmok@alumni.ubc.ca (J.C.S.); andyliu@ece.ubc.ca (M.-C.L.); lukasc@ece.ubc.ca (L.C.)

**Keywords:** spatiotemporal microfluidic oxygen control, optical oxygen sensors, transient/cyclic hypoxia

## Abstract

Control of oxygen over cell cultures *in vitro* is a topic of considerable interest, as chronic and cyclic hypoxia can alter cell behaviour. Both static and transient hypoxic levels have been found to affect tumour cell behaviour; it is potentially valuable to include these effects in early, *in vitro* stages of drug screening. A barrier to their inclusion is that rates of transient hypoxia can be a few cycles/hour, which is difficult to reproduce in traditional *in vitro* cell culture environments due to long diffusion distances from control gases to the cells. We use a gas-permeable three-layer microfluidic device to achieve spatial and temporal oxygen control with biologically-relevant switching times. We measure the oxygen profiles with integrated, ratiometric optical oxygen sensors, demonstrate sensor and system stability over multi-day experiments, and characterize a pre-bleaching process to improve sensor stability. We show, with both finite-element modelling and experimental data, excellent control over the oxygen levels by the device, independent of fluid flow rate and oxygenation for the operating flow regime. We measure equilibration times of approximately 10 min, generate complex, time-varying oxygen profiles, and study the effects of oxygenated media flow rates on the measured oxygen levels. This device could form a useful tool for future long-term studies of cell behaviour under hypoxia.

## 1. Introduction

Hypoxia, or low oxygen levels, is often present in tumours, and oxygen levels in tumours can vary in both time and space. Because the presence of hypoxia can affect cancer prognosis and treatment efficacy, understanding the effects of hypoxia in early, *in vitro*, stages of testing cancer treatments could help in the development of effective treatments. For cell-based high throughput screening, relevant tissue constructs [1] should accurately reproduce hypoxic profiles that can exist in tumours, including chronic hypoxia, oxygen gradients, and cyclic or transient hypoxia. Accomplishing this requires a platform that affords the creation of oxygen profiles that vary in time and space, precise control of the oxygen levels, and stability over time for long-term cell culture (as conventional drug screening assays can last several days).

### 1.1. Importance of Precise Oxygen Control in Hypoxia Studies

Compared to physiological oxygen levels of approximately 10% [2], approximately 60% of solid tumour mass is either hypoxic or anoxic [3] and the degree of hypoxia has been associated with poor prognosis [4] and tumour aggressiveness. Based on binding of the 2-nitroimidazole agent EF5 to resected tissue, Evans *et al.* [5] calculated tissue oxygen partial pressure (pO_2_) and presented five categories to describe oxygen levels within gliomas: physiologic (10%), modest hypoxia (2.5%), moderate hypoxia (0.5%), severe hypoxia (0.1%) and anoxia (0%). Grade 2 tumours on average had modest hypoxia, grade 3 tumours had moderate hypoxia, and grade 4 tumours had severe hypoxia; increased levels of hypoxia were associated with more rapid tumour recurrence.

Hypoxia has been found to increase tumour malignancy by developing cells with more aggressive phenotype [6], and by upregulating genes associated with breast cancer metastasis [7,8]. Two regulatory cascades have been investigated as targets for anticancer therapy: the hypoxia-inducible-factor (HIF) pathway is involved at hypoxia levels around 1% O_2_, and the unfolded protein response, which operates at hypoxia levels <0.2% O_2_ [8,9]. While investigating hypoxia-induced breast cancer cell migration in the unfolded protein response pathway, Nagelkerke *et al.* [10] found that hypoxia of 1% O_2_ stimulated more migration of MDA-MB-231 breast cancer cells than 0.5% O_2_ in a gap closure assay. In contrast, at severe hypoxic conditions of 0.1% O_2_ the cell migration was not statistically significant compared to normoxic control, which may have indicated that cell survival was more important than cell migration under those conditions. Severe hypoxia (0.1%) was also found to activate different levels of genes compared to 1% hypoxia [11]. It has also been found that the degree of hypoxia can affect autophagy in breast cancer cells: after inducing hypoxia in MCF-7 cells using hypoxic incubation, Bellot *et al.* [12] measured the increase in GFP-LC3 puncta within cells and the number of autophagic vacuoles and showed that severe hypoxia (0.1% O_2_) induced increased autophagy compared to cells cultured under moderate hypoxia (1% O_2_).

These results suggest that precise oxygen control is highly beneficial when developing platforms to study specific hypoxic regimes that can exist in tumours. Beyond cancer research and tumour cell culture, this type of precise oxygen control could also have other biological applications. The degree of hypoxia has also been found to be important in regulating embryonic development [13] and stem cell function [14], indicating that hypoxia control platforms could have wider biological relevance.

### 1.2. Clinical Relevance of Oxygen Gradients and Cyclic Hypoxia

In addition to chronic, static hypoxic conditions, there is also evidence that spatially varying oxygen gradients as well as temporally varying oxygen levels can exist in tumours. To measure oxygen gradients, Helmlinger *et al.* used phosphorescence lifetime imaging to image oxygen profiles in 27-day old human colon adenocarcinoma tumours in immunodeficient mice. They measured gradients near blood vessels decreasing from 14 to 0 mmHg (1.8% to 0%) over approximately 200 μm [15]. Another work imaged oxygen gradients within mammary adenocarcinomas in rats using phosphorescence lifetime imaging through dorsal skin flap window chambers, and measured gradients between approximately 1–35 mm Hg (0.1%–4.6%) under air breathing conditions [16].

While chronic or prolonged hypoxia has been found to act as a selective pressure for cells that are resistant to apoptosis [17,18], intermittent or cyclic hypoxia can also select for genotypes leading to tumour progression and resistance to therapy [19]. Large fluctuations in blood flow and oxygenation within tumours have been seen in animal tumour models [10,20,21] and human tumours [7,22,23]. The cycling frequency can range from several cycles per hour to cycles in days [24].

### 1.3. Harnessing Small Size Scales: Microfluidic Oxygen Control with Optical Oxygen Sensing

#### 1.3.1. Microfluidic Oxygen Control Devices

Microfluidic devices are attractive for oxygen control due to the level of spatial as well as temporal control that they can facilitate. The permeability of poly (dimethylsiloxane) (PDMS) microfluidic devices to oxygen is very high, which permits the use of separate channels (separated by thin PDMS membranes) to control the oxygen levels. Additionally, the diffusion distances between the channels in a microfluidic device can be very small. These short diffusion distances lead to fast equilibration times, which facilitate the creation of time-varying oxygen profiles with fast switching times, recreating the kinds of switching times of several cycles per hour that have been seen in animal tumour models.

Several groups have presented devices to make spatial gradients in oxygen levels in a microfluidic device, using gases [25,26,27], gas-equilibrated liquids [28,29], and oxygen-generating and oxygen-scavenging chemicals and chemical reactions [30,31]; a review on oxygen control using microfluidic platforms summarizes the various methods that have been employed [32]. One potential complication of the use of pressurized gases to control the oxygen levels is the tendency for bubbles to form in the cell culture channel, or for the media in the cell culture channel to evaporate through the thin PDMS membrane. A solution to this issue is the use of gas-equilibrated liquids as the control media; however, accurate control necessitates the use of very high flow rates that are impractical to provide via syringe pump. Additionally, the time necessary to oxygenate/deoxygenate these fluids adds to the equilibration time of the system, precluding the possibility of creating fast switching times within the device. As another solution to the problem of evaporation and bubble formation, Wood *et al.* have reported the use of a hydration layer between the gas-containing control layer and the sample [33].

#### 1.3.2. Optical Oxygen Sensors

In many of these microfluidic oxygen control devices, optical oxygen sensors, operating on the principle of the reversible quenching of luminescence intensity and lifetime, were used to measure the oxygen profiles created in the device due to their ability to be easily miniaturized and measured optically (not requiring electrical connections), as well as because they do not consume oxygen [34,35]. Intensity-based sensors are the most straightforward to measure, requiring only a fluorescence microscope; however, the luminescence intensity is also affected by a number of other factors such as inhomogeneities in the measurement system and sensor film, as well as photobleaching and dye leaching [36]. Lifetime-based sensors are far more robust but require a more complicated measurement setup with fast, time-resolved imaging [37]. Ratiometric sensors, which use a reference (oxygen-insensitive) luminescent dye within the sensor matrix to compensate for some inhomogeneities in the measurement system, offer enhanced robustness and stability while only requiring a fluorescence microscope for measurement [38,39]. Ungerböck *et al.* have presented a thin-film ratiometric sensor composed of platinum(II)-5,10,15,20-tetrakis-(2,3,4,5,6-pentafluorphenyl)-porphyrin (PtTFPP) and Macrolex Fluorescent Yellow (MFY), wherein the MFY acts as both the reference dye and an antenna dye (using light harvesting [40] to help excite the PtTFPP by transferring energy) [41,42]. The luminescence of these films can be spectrally resolved using the colour channels of a colour camera, making them a convenient sensing platform offering improved robustness over intensity-based sensors with fluorescence microscope readout. These ratiometric sensors have been found to follow a simplified two-site quenching model:

(1) $$\frac{R}{R_{0}}=\frac{f_{1}}{1+k_{SV}pO_{2}}+f_{2}$$

where *R* and *R*_0_ are the ratiometric intensities (the ratio of the oxygen-sensitive channel’s fluorescence intensity to that of the reference channel: *R* = *I_sens_*/*I_ref_*) in deoxygenated conditions and under oxygen partial pressure *pO*_2_, respectively; *k_SV_* is the Stern-Volmer quenching constant (a measure of the quenching efficiency); and *f*_1_ and *f*_2_ describe the fraction of the dye molecules that is quenched by oxygen with Stern-Volmer quenching constant equal to *k_SV_*, and the fraction of dye molecules that are non-quenchable (Stern-Volmer quenching constant equal to 0), respectively [42]. A similar concept has since been expanded by the same group to perform ratiometric imaging of both oxygen and pH [43].

#### 1.3.3. Microfluidic Devices for Cyclic (Transient) Oxygen Profiles

Microfluidic systems have also been used to create cyclic oxygen profiles. Groisman *et al.* presented a microfluidic device with integrated spatiotemporal control of oxygen levels, demonstrating oxygen switching times on the order of seconds within a thin, multilayer microfluidic device with a gas control layer [44]. Oppegard *et al.* presented a microfluidic insert containing control gas channels for a 6-well plate to modulate the oxygen levels over adherent cell cultures, demonstrating equilibration times on the order of minutes [45]; the same group used a similar device to control the oxygen levels within a Boyden chamber, showing equilibration times of approximately 20 min [46]. Both of these works showed impressive spatiotemporal control of the oxygen levels, stable oxygen levels over several days, as well as biological validation of the devices. However, there remained potential for media evaporation through the PDMS membrane and into the flowing gas used to control the oxygen levels during multi-day culture in these devices (the authors took steps to pre-hydrate the PDMS to discourage evaporation through it, mitigating this effect for shorter term experiments). To measure the oxygen levels, intensity-based optical oxygen sensors were used, which can suffer from instabilities over long-term experiments due to fluctuations in the fluorescence excitation intensity as well as due to the effects of photobleaching or dye leaching. Similar oxygenators employing microfluidic gas control channels have also been applied to spatiotemporal control of oxygen over *ex vivo* pancreatic islets (1 min cycles of 5% and 21% oxygen for one hour) [47] and brain slices (spatial oxygen control to expose part of the slice to hypoxia, as well as temporal oxygen control with equilibration times of ~10 min) [48]; both of these works used non-integrated commercial probes to measure the oxygen levels. Another work used a microfluidic device, again with gas control channels, to expose cardiomyocytes to different transient hypoxic profiles and measure the oxygen levels using a water-soluble optical ruthenium-based probe, showing a dependence of steady-state oxygen level on liquid flow rates [49]. Wood *et al.* used a three-layer microfluidic device to study the effects of deoxygenation on red blood cell velocity under constant pressure, using a hydration layer between the gas and liquid to prevent blood dehydration and using a commercial oxygen probe to measure oxygen levels in the gas phase during the experiment [33].

Here, we report on the development of a microfluidic system for spatial and temporal oxygen control and measurement with the goal of long-term 3-D perfusion cell culture and monitoring. Our three-layer microfluidic device contains individually-addressable gas control channels on three sides of the cell culture chanel, as well as liquid-perfused hydration channels between the gas and cell culture channels to reduce bubble formation and evaporation in the cell culture channel. We integrate ratiometric optical oxygen sensors into the device that can map oxygen levels in 2-D, and demonstrate their improved stability over intensity sensors.

## 2. Experimental Section

### 2.1. Fabrication of Optical Oxygen Sensors and Microfluidic Oxygen Control Device

#### 2.1.1. Design of Oxygen Control Device

**Figure 1:**
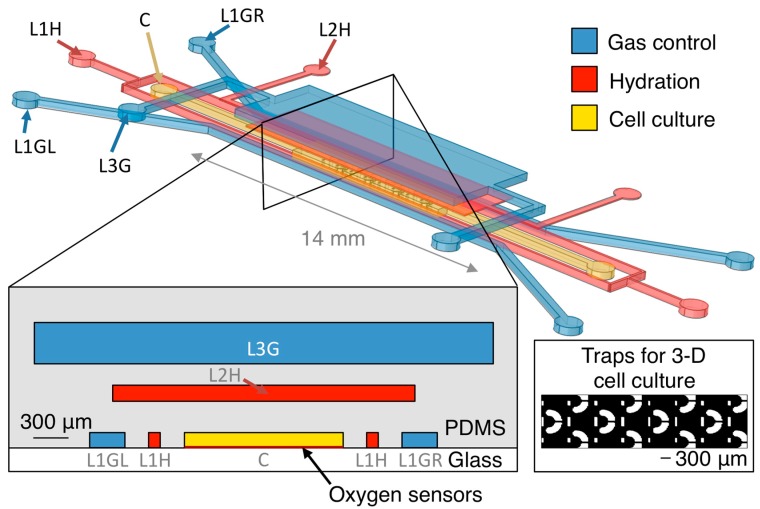
Oxygen control microfluidic device layout, showing 3-D and cross-sectional views of the device as well as the position of the integrated optical oxygen sensors within the cell culture channel. The cell culture channel (C) is surrounded on three sides by gas control channels (L1GL, L1GR, and L3G), with hydration/bubble removal channels (L1H, L2H) between each of the gas channels and the centre channel. Inset shows trapping structures for 3-D cell cultures within the cell culture channel. Please note that the 3-D view is not to scale (microfluidic layers are spaced farther apart in the illustration than in the device to permit visualization of the three layers). Cross-sectional view is to scale.

The oxygen control microfluidic device is designed to support either 2-D or 3-D cell culture in its centre channel. Our group uses microfluidically-generated hydrogel beads containing cells that can proliferate into spheroids for 3-D cell culture and drug screening [50,51], so we have designed our device to have 300 μm hydrodynamic trapping structures (with a small flap in front of the trap opening to aid in trap retention under small amounts of backflow [52]) to immobilize the spheroids in the device for monitoring. The cell culture channel (1.35 mm × 7 mm) within the tested device contains 12 trapping structures as depicted in the inset of Figure 1, which permit the immobilization of 12 cell-laden hydrogel beads for 3-D cell culture, each of 300 μm diameter; the size and design of the cell culture channel could also be tailored to permit the culture of different sized 3-D spheroids or more cells in 3-D or monolayer culture. Our multilayer microfluidic oxygen control device is depicted in Figure 1.

The device consists of three layers of channels in PDMS on a glass substrate. A centre channel for cell culture on the first layer of the device (C), is surrounded on three sides by oxygen control gas channels (L1GL and L1GR on the left and right, respectively, of the cell culture channel on layer 1, and L3G on layer 3). There is a 1.3 mm entrance region at the start of the cell culture chamber before trapping structures begin to permit gas equilibration with the fluid before it reaches the cell cultures; the size of this region was estimated based on oxygen gradient measurements from previous design iterations of the oxygen control device. Gas channels are individually addressable to permit the creation of oxygen gradients within the device. Additional fluid-containing channels similar to the hydration layer previously presented by Wood *et al.* [33] (L1H on layer 1 and L2H on layer 2) are integrated between the cell culture channel and the gas channels to discourage sample evaporation from and bubble formation within the cell culture channel, which are potential disadvantages of the use of pressurized gases for oxygen control [28].

#### 2.1.2. Fabrication of Oxygen Control Device

The 3-layer device was fabricated by standard multilayer soft lithography using SU-8 3050 photoresist (Microchem, Westborough, MA, USA) on silicon masters, with an intermediate polyurethane mould for the thick (layer 3) portion. Layers were bonded by air plasma bonding; layers were exposed to air plasma (Harrick plasma cleaner) for 75 s before bringing the layers into contact, pressing gently, and leaving the device in an oven at 70 °C for 30 min. After demolding, inlets and outlets were punched and the piece was bonded to the next layer or integrated with the sensor-containing substrate. After bonding the PDMS layers but before integrating with the glass substrate, several devices from the same fabrication batch as the tested samples were sacrificed, diced near the centre and end of their channels, and examined using microscopy to measure channel and membrane heights. The layer 1 channel height was measured to be 130 ± 6 μm, the PDMS membrane between layers 1 and 2 was measured to be 260 ± 30 μm, the layer 2 channel height was 137 ± 7 μm, the membrane between layers 2 and 3 was 174 ± 50 μm, and the layer 3 channel height was 350 ± 30 μm.

#### 2.1.3. Fabrication of Optical Oxygen Sensors and Integration with Microfluidic Oxygen Control Device

Ratiometric optical oxygen sensors similar to those described by Ungerböck *et al.* [42] were fabricated using a laser-cutting patterning method developed by our group [53]. Briefly, polystyrene films containing 1% *w*/*w* PtTFPP (Frontier Scientific, Logan, UT, USA) and 2% *w*/*w* MFY (LANXESS, Cologne, Germany) films were formed. The dyes were first dissolved in a 5% solution of polystyrene in toluene, and the solution was mixed using a magnetic spin bar for 1–2 h as well as sonicated for >30 s in an effort to ensure dye distribution uniformity within the solution. After mixing, the solution was spin-coated (2000 RPM, 1 min) on glass microscope slides that had been dehydration-baked at 125 °C and pre-treated with a 5-min exposure to oxygen plasma (400 mTorr, 160 W). The toluene was then left to evaporate for 5–10 min before the sensors were baked on a hotplate for 5 min at 125 °C. A laser cutter was then used to cut around the edges of the sensor patches, and the film bulk was lifted off with the aid of a water bath (taking advantage of the relatively poor adhesion of polystyrene films to glass surfaces), leaving behind the isolated sensor patches. This liftoff process was used because we have previously found that residues left after plasma etching of polystyrene films can inhibit plasma bonding of PDMS microfluidics to the glass substrate; further details on the laser patterning process and results are presented elsewhere [53]. Sensors were then baked on a hotplate for 5 min at 115 °C after liftoff but before integration with the microfluidic device. The optical oxygen sensors were integrated with the microfluidic oxygen control device via irreversible plasma bonding with the glass substrate, using the same air plasma bonding method described in Section 2.1.2, used to bond the layers of the multilayer device.

### 2.2. Measurement Setup for Oxygen Sensors

The sensors were measured within a setup composed of a custom gas mixing and supply system and a fluorescence microscope (TE2000U, Nikon Canada, Mississauga, ON, Canada). A mercury arc fluorescence excitation source (Intensilight, Nikon Canada, Mississauga, ON, Canada) passed through a fluorescence filter block (XF115-2, Omega Optics, Brattleboro, VT, USA) to excite the sensor films. A custom software GUI written in C# using the .NET framework communicated with the camera (Retiga EXi, Qimaging, Surrey, BC, Canada), microscope stage controller (ProScan II, Prior Scientific, Rockland, MA, USA), and custom-built shutters. The shutters were fitted onto the microscope to selectively block the fluorescence and brightfield light sources, permitting long-term automated imaging of the samples. This software GUI also communicated with a microcontroller (Arduino Mega, Arduino, Torino, Italy) controlling an array of solenoid valves to mix known proportions of air, nitrogen, and carbon dioxide, store the mixed gases in small storage tanks, and supply them to the microfluidic system (switching between them in an automated fashion to create automated time-varying oxygen profiles within the device).

The device, immersed in water during all fluidic oxygen measurements, remained in a microscope stage-top incubator controlled to 37 °C for all calibrations and measurements. All connections from the chip to the inlet and outlet tubes (Tygon micro-bore tubing, Cole-Parmer Canada, Montreal, QC, Canada) were made via friction fitting of 22 gauge blunt needles (Nordson EFD, Westlake, OH, USA) into holes cored into the PDMS using a 0.5 mm biopsy punch (Harris Uni-Core, Ted Pella, Redding, CA, USA). A syringe pump (Chemyx, Stafford, TX, USA) was used to control the flow rate in all liquid-containing channels.

### 2.3. Calibration of Optical Oxygen Sensors

#### 2.3.1. Sensor Calibration Setup

After fabrication and integration with the microfluidic device, the oxygen sensors were calibrated for both gaseous and fluidic oxygen measurements. Sensors were first calibrated for gaseous oxygen sensing by flowing gas containing known oxygen levels (measured with a commercial oxygen sensor: Fibox 2, Presens, Regensburg, Germany) through the cell culture channel, directly over top of the sensors. For fluidic calibration, the cell culture and hydration channels (C, L1H, L2H) were filled with water, and gas was supplied to the gas control channels (L1GR, L1GL, L3G) as well as the environment around the chip. Fluid flow in the liquid-filled channels was stopped using pinch clamps (Cole-Parmer Canada, Montreal, QC, Canada) on the micro-bore tubing inlets, and all calibration points were acquired after equilibrium was reached (images were acquired every 30 s after gas supply began, and this image series was used to determine equilibration).

#### 2.3.2. Image Processing

For both gaseous and fluidic calibration as well as subsequent measurements, 3 images at each datapoint were acquired and averaged. The ratiometric intensity R was calculated from the ratio of the red to the green colour channels of the colour camera (as the PtTFPP emits in the red channel while MFY emits in the green). The averaged R_0_/R result for all pixels in each sensor patch at each gas level was plotted *vs.* the oxygen level and fitted to the simplified two-site quenching model (Equation (1)). For the purposes of showing the calibration plots, the responses from four sensor patches were averaged. For subsequent measurements using the sensors, each sensor was measured using its own calibration parameters (*i.e.*, sensor-by-sensor calibration was performed and used).

### 2.4. Finite-Element Modeling of Oxygen Control Device

The oxygen control microfluidic device was simulated using COMSOL^®^ Multiphysics. The Navier-Stokes equations were solved to find the fluid velocities in the gas and liquid phase channels. For the gas phase channels (L1GR, L1GL, L3G in Figure 1), compressible turbulent flow was simulated, based on the approximated Reynolds Number (27,000). The gas inlet relative pressure boundary conditions were defined as 1000 Pa, based on measurements of the pressure at the chip inlet by a pressure sensor (HDI, First Sensor, Berlin, Germany) and the outlet pressure boundary conditions defined at 0 Pa (with an absolute pressure of 101,325 Pa). Wall boundaries were defined as wall functions with no wall roughness.

For the liquid phase channels (C, L1H, L2H in Figure 1), incompressible laminar flow was simulated. The inlets were defined as velocity-based boundary conditions based on the volumetric flow *Q* (*v* = *Q*/*A*, where *A* is the cross-sectional area of the channel inlet). Various liquid flow rates were simulated. The outlets were defined as pressure boundary conditions (0 Pa), and all other walls defined as no-slip boundaries.

**Figure 2:**
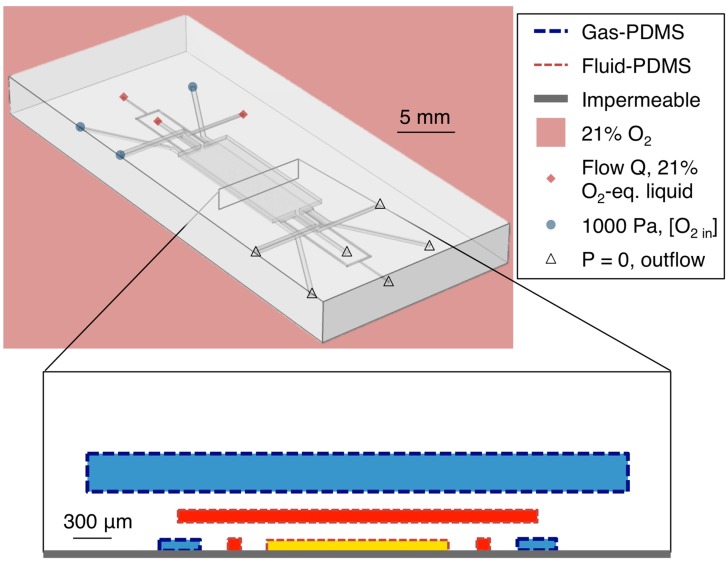
Simulation geometry and boundary types. Simulated channel heights were 100 μm for layers 1 and 2 (C, L1H, L1GL, L1GR, L2H), and 300 μm for layer 3 (L3G). Simulated PDMS membrane thicknesses were 100 μm both between layers 1 and 2 and between layers 2 and 3.

To simulate the oxygen levels within the device, three instances of the convection-diffusion equation were solved (one for the oxygen concentration in the gas phase, one for that dissolved in PDMS, and one for that dissolved in media/water). The velocity fields in the gas and liquid phases were taken as the results of the flow modeling described above. Boundary conditions and values for the diffusivity (*D*) and solubility (*S*) of oxygen in PDMS and cell culture media (water) were based upon Kim *et al.*’s previous work on mathematical modeling of oxygen transport in microfluidic devices (*D_PDMS_* = 7.88 × 10^−5^ cm^2^/s; *D_H2O_* = 2.8 × 10^−5^ cm^2^/s; *S_PDMS_* = 1.25 mM/atm; *S_H2O_* = 0.218 mM/atm) [54]. A schematic showing the simulation boundary types and locations in the device layout is presented in Figure 2.

The boundaries at the bottom surface of layer 1 (in contact with the glass in the fabricated device) were simulated as oxygen-impermeable. At the gas-PDMS boundaries within the device, Henry’s Law was used to relate the concentrations of oxygen in the gas phase and in the PDMS (*c_g_* and *c_PDMS_*, respectively):

(2) $$c_{PDMS}=S_{PDMS}\cdot pO_{2}\approx S_{PDMS}\cdot c_{g}\cdot R\cdot T$$

where *pO*_2_ represents the partial pressure of oxygen, *R* the ideal gas constant, and *T* the temperature. At the PDMS-water boundaries, *pO*_2_ was taken to be constant and Henry’s Law again applied:

(3) $$\frac{c_{PDMS}}{S_{PDMS}}=\frac{c_{H2O}}{S_{H2O}}$$

Gas inlet oxygen concentrations were simulated based on experimental conditions; boundary values ranged from 0% to 10% oxygen and are noted in the results section for each simulation. All channel outlets were set to outflow boundary conditions. For all simulations, the exterior of the PDMS chip as well as the fluid at each liquid inlet was simulated as exposed to air (21% oxygen).

The independence of the oxygen levels in the device from the atmospheric condition, and from the oxygenation of the input fluid, was simulated at steady-state using a stationary solver. Additionally, the equilibration times in the device after changing the gas control input were simulated by running a time-dependent solver. For all simulations, different maximum and minimum mesh cell sizes (ranging from minimum 1.34 μm and maximum 20 μm in the hydration channels to minimum 27 μm to maximum 250 μm in the PDMS bulk) were specified for the different domains within the device (the five channel regions and the PDMS bulk). Free tetrahedral meshing optimized for fluid dynamics was used in all cases, with a total of 1.3 × 10^7^ domain mesh elements, 5.3 × 10^5^ boundary mesh elements, and 2.4 × 10^4^ edge mesh elements. We found that increasing the domain mesh elements to 2.2 × 10^7^, the boundary mesh elements to 8.2 × 10^5^, and the edge mesh elements to 3.1 × 10^4^ resulted in a change in the simulated average oxygen level in the cell-containing channel region of less than 1% of the average value, suggesting that our simulations were acceptably mesh independent.

### 2.5. Measurements of Oxygen Equilibration Times, Time-Varying Oxygen Levels and Oxygen Gradients

In order to prevent gas dissolved in the PDMS from entering the microfluidic channels and forming bubbles when the chip was brought up to 37 °C, a pre-hydration/degassing protocol (used by our group for all on-chip cell culture experiments) was employed. Before use, the PDMS microfluidic devices were immersed in water and degassed using a desiccator and vacuum pump for >1 h. Devices in water were then brought to 70 °C by placing them in an oven for several hours in a covered beaker. The liquid was then partly cooled (bringing the devices to approximately 40 °C) and a desiccator was again used to further degas the chips for >1 h. Liquids supplied to the chip were treated with the same protocol.

After sensor calibration (discussed in Section 2.3), the automated gas system (discussed in Section 2.2) was used to supply known oxygen levels to the gas control channels. For measurements of oxygen equilibration times and time-varying oxygen levels, the same gas was input to all three gas channels and switched at defined time points. For oxygen gradient measurements, a different gas was supplied to the L1GL from that supplied to the L1GR and L3G channels of the device. Images of the device (in brightfield) and sensors (fluorescence) were acquired every 60 s. For the measurements of equilibration and time-varying profiles, the average oxygen level in the sensor regions was plotted *vs.* time. For the gradient measurements, the time-series was first plotted to determine when equilibration had been reached (approximately 10 min) and then the oxygen measurements from the sensors in an image after equilibration were averaged (the pixels in three sensors along the channel length) to plot the oxygen gradient *vs.* channel width position. For all measurements of equilibration times, time-varying oxygen levels, and oxygen gradients, water was continuously perfused through the C, L1H, and L2H channels at a rate of 0.5 μL/min using a syringe pump.

## 3. Results and Discussion

### 3.1. Calibration Results and Sensor Limits of Detection

**Figure 3:**
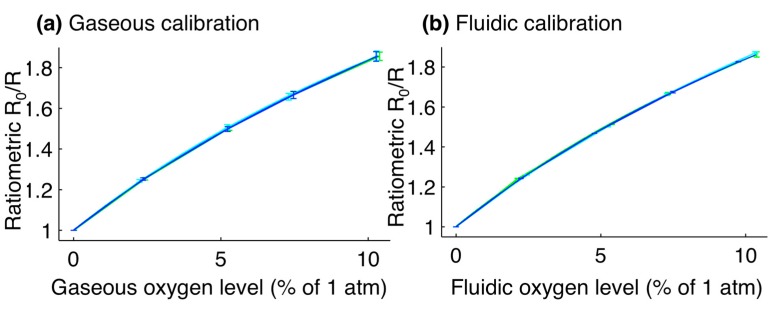
Calibration plots for optical oxygen sensors. (**a**) Gaseous calibration, with fit parameters of: k_sv_ = 0.151% ± 0.004% atm^−1^; f_1_ = 0.756 ± 0.007; f_2_ = 0.243 ± 0.007; (**b**) Fluidic calibration, with fit parameters of: k_sv_ = 0.152% ± 0.006% atm^−1^; f_1_ = 0.755 ± 0.016; f_2_ = 0.243 ± 0.015. For the fluidic calibration, the oxygen level is reported as the level of the gas in equilibrium with the liquid. Error bars represent the standard deviation in ratiometric intensity between four different sensor patches. Line colours represent the calibration curves for three different repetitions of the calibration.

The results of the gaseous and fluidic calibration of the sensors are presented in Figure 3. Fit parameters for the gaseous calibration were calculated (the average and one standard deviation error of three repeats of the calibration, each on the averaged response from four sensor patches) as k_sv_ = 0.151% ± 0.004% atm^−1^; f_1_ = 0.756 ± 0.007; f_2_ = 0.243 ± 0.007. Fit parameters for the fluidic calibration were calculated (three repeats of the calibration on the averaged response from four sensor patches) as k_sv_ = 0.152% ± 0.006% atm^−1^; f_1_ = 0.755 ± 0.016; f_2_ = 0.243 ± 0.015. These constants are similar to those previously reported by Ungerböck *et al.* (k_sv_ = 11.5 × 10^−3^ hPa^−1^ = 0.1165% atm^−1^; f_1_ = 0.785; f_2_ = 0.215).

The Limit of Detection (LoD) for the sensors was calculated based on the methods described by Armbruster and Pry [55]. First, the Limit of Blank (LoB) was determined; the LoB is defined as the highest oxygen level expected to be measured by the system when zero oxygen is present [55]. It was calculated by exposing the sensors to zero oxygen (directly to gas for the gaseous LoDs, and via the microfluidic oxygen control chip for fluidic calibration) and acquiring >5 readings from the integrated sensors. The LoB was then calculated as 95% of these measured values assuming a Gaussian distribution [55]: LoB = mean_blank_ + 1.645(SD_blank_).

This process was repeated for a sample containing 2% oxygen in order to find the LoD, which is defined as the lowest oxygen level that can be measured by the system and discerned from the LoB [55]. The LoD was calculated from the LoB and the standard deviation of the measurements taken as 2% oxygen was supplied to the system, again assuming a Gaussian distribution and finding a 95% confidence interval [55]: LoD = LoB + 1.645(SD_sample_). All datapoints were acquired after equilibrium was reached, and LoDs reflect the stability of the full system (gas supply system, microfluidic oxygen control device, and sensors). Each LoD was calculated by averaging the LoDs of four sensor patches and two sets of measurements (N = 8), each calculated from >5 measurements. The calculated LoDs were 0.06% for the gaseous oxygen levels and 0.08% for fluidic oxygen levels. These LoDs are sufficiently precise to distinguish between modest (2.5%), moderate (0.5%), and severe (0.1%) hypoxia. In order to improve the sensor LoD, and particularly for studies at very low oxygen levels below 0.1%, future work could include the investigation of different oxygen-sensitive indicators with higher sensitivities; either PdTFPP, which has approximately twice the sensitivity of PtTFPP [56,57], or other ultra-sensitive sensors [58], could be employed.

### 3.2. Improvement of Ratiometric Oxygen Sensor Stability by Pre-Bleaching and Multiple Calibrations

To test the sensors’ stability during long-term experiments, an analysis of the ratiometric intensity over time was performed. First, the oxygen levels measured with the ratiometric method were compared to those obtained from the intensity alone during a fluidic experiment. After fluidic calibration, the oxygen levels supplied to the gas control channels were switched between 0%, 2%, and 7% oxygen over a period of 2 h while the cell culture channel was perfused with water at a rate of 0.5 μL/min. The device was imaged every 60 s during the course of this experiment using an excitation shutter (providing approximately 3 s of exposure every 60 s) and the oxygen levels were calculated from both the ratiometric signal and the intensity signal alone, and compared.

During long experiments where an excitation shutter was not used, we observed that measured oxygen levels drifted upwards over time, indicating that the ratiometric intensity decreased over time. This observation was consistent with sample photobleaching, with the PtTFPP bleaching at a faster rate than the MFY. Indeed, the measured intensities of the red and green channels of the sensor images showed that the luminescence intensities of both dyes decreased over time under continuous exposure. This effect was observed during both gaseous and fluidic oxygen measurements, suggesting that photobleaching, rather than dye leaching, was the dominant effect. We have investigated two methods of improving the sensor stability over time: the use of the excitation shutter to limit sensor exposure to the excitation light (which will also be necessary for subsequent cell-based studies using the system) and pre-bleaching of the sensor films to decrease the rate of ratiometric intensity decrease over time. To test the pre-bleaching procedure, sensors, within the microscope stage top incubator controlled to 37 °C and exposed to air (21% O_2_), were imaged every 10 min over time periods greater than 12 h. Sensors were imaged using the microscope shutter (yielding approximately 3 s of exposure every 10 min), as well as without the shutter (yielding constant exposure).

**Figure 4:**
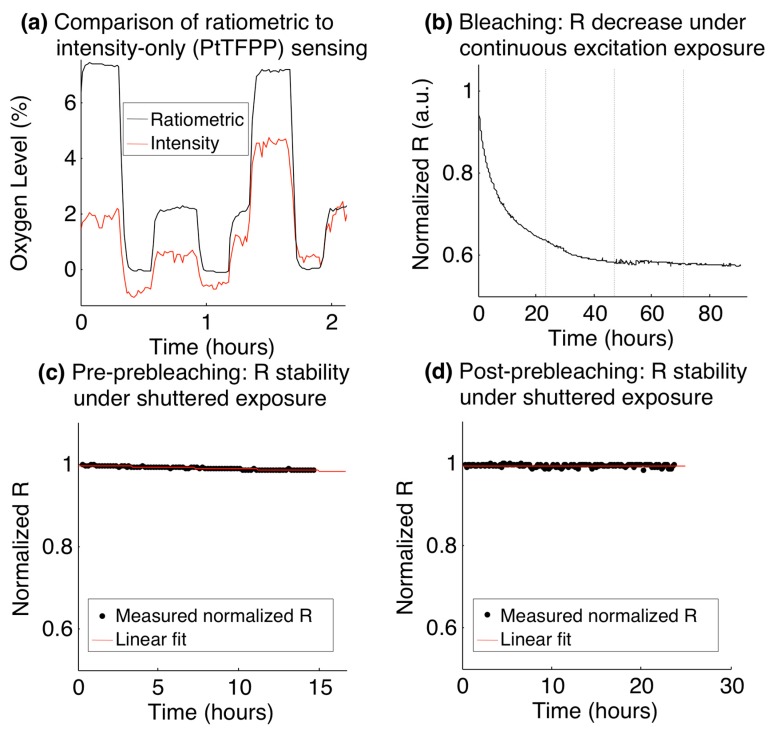
Stability of ratiometric sensors over time. (**a**) Comparison of ratiometric stability with the stability of the PtTFPP channel alone during a fluidic oxygen switching experiment; (**b**) Ratiometric intensity decrease over time while being constantly exposed to the fluorescence excitation source (dotted vertical lines denote increments of days); (**c**) Stability of the ratiometric intensity (black), before pre-bleaching. The standard deviation of the normalized ratiometric intensity over the 14.5 h time course was 0.0038, and a linear fit to the normalized ratiometric intensity (red) shows a slope of −1.5 × 10^−5^ min^−1^; (**d**) Stability of the ratiometric intensity (black), after a 61-h pre-bleaching process. The standard deviation of the normalized ratiometric intensity over the 23.5 h time course was 0.0035, and a linear fit to the normalized ratiometric intensity (red) shows a slope of −1.4 × 10^−6^ min^−1^.

A summary of the results of the ratiometric stability experiments is presented in Figure 4. Figure 4a presents a comparison between the oxygen levels measured by the ratiometric method to those measured by intensity alone from the same sensor (PtTFPP channel only), during the fluidic oxygen switching experiment. The ratiometric measurements show a much more stable profile, consistent with previous literature on ratiometric sensors. We believe that the dominant cause for the observed intensity fluctuations is the mercury arc lamp excitation source used in many fluorescence microscopes. These results suggest that ratiometric sensors are far better suited to long-term experiments than the intensity-based sensors employed in some of the previous demonstrations of microfluidic cyclic hypoxia.

Figure 4b–d present the stability over time of the ratiometric intensity (R), normalized to its value at the start of the experiment, as the sensor was left exposed to 21% O_2_ atmospheric conditions and controlled to 37 °C. Figure 4b presents the observed decrease in normalized R during an experiment in which the sensor film was left constantly exposed to the mercury arc lamp excitation source. The curve sharply decreases over the first 24 h and approaches a stable value close to 60% of its initial ratiometric intensity, levelling off after approximately 48 h.

With this behaviour in mind, we tested a prebleaching process in which the ratiometric stability was analyzed both before and after 61 h of constant exposure to the excitation source (prebleaching). In each test, the sensor was imaged with the use of the fluorescence excitation shutter (which yielded approximately 3 s of exposure every 10 min imaging cycle) for periods >12 h. Figure 4c,d present the results of this study, with Figure 4c depicting the ratiometric stability before the pre-bleaching process, and Figure 4d depicting the stability after pre-bleaching. The data were fitted to a line to analyze the rate of decrease of the ratiometric intensity in each case, and the standard deviation of all of the measurements in the time series was taken as a measure of the stability in each case.

The slopes before and after the pre-bleaching process were found to be −1.5 × 10^−5^ min^−1^ and −1.4 × 10^−6^ min^−1^, respectively, showing a decrease of over an order of magnitude in the rate of ratiometric intensity attenuation after the pre-bleaching process. The disadvantage of the pre-bleaching process is the overall luminescence intensity degradation that it induces, which leads to noisier signals after the bleaching process. For long experiments, however, this additional noise will be counteracted by the decrease in the downward slope of R over time. In our study, we found that the standard deviation of the normalized ratiometric intensity measurements over the 14.5 h time course of the experiment before pre-bleaching was 0.0038, and that over the 23.5 h time course of the experiment after the pre-bleaching was 0.0035. The stability of the measurements was thus greater over the longer experiment after pre-bleaching than that during the shorter experiment before, even with the increased noise induced by the lower intensities after bleaching. The effect of the slope in the ratiometric intensity would only become more pronounced during longer experiments, while the noise induced after pre-bleaching could be reduced at oxygen levels of physiologic relevance (less than the 21% used in this experiment), due to the reduction in quenching of the PtTFPP luminescence.

### 3.3. Validation of Oxygen Control within the Device Using Finite-Element Modeling

**Figure 5:**
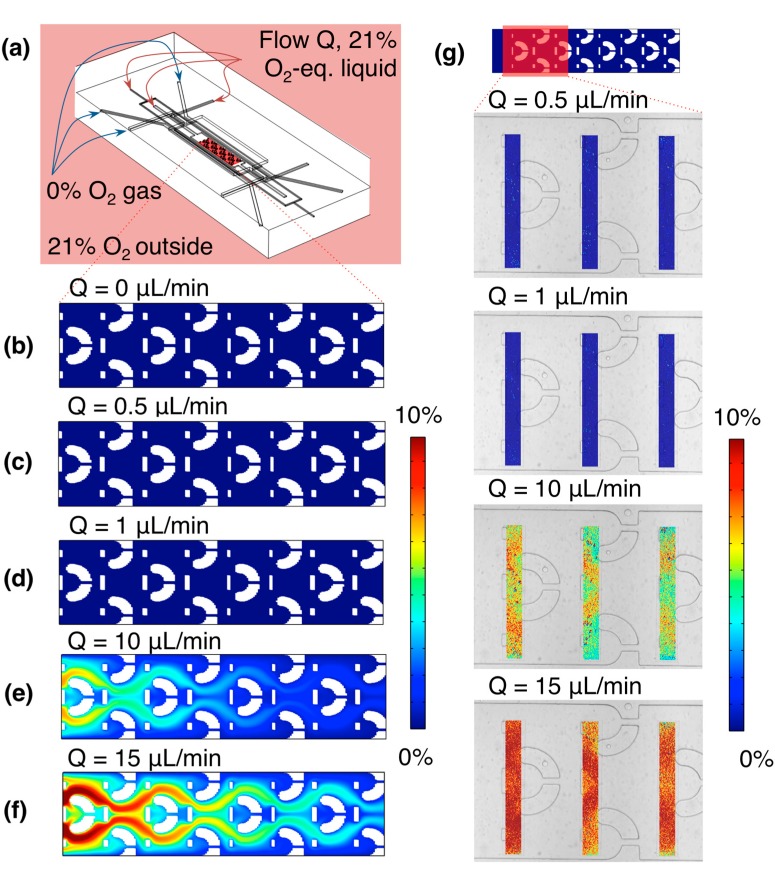
Simulated and experimental oxygen levels within the microfluidic device, at various rates of fluid flow in the cell culture channel and bubble removal/hydration channels (the same rate supplied to all three channels). (**a**) Simulation geometry, showing the region analyzed (a 2-D slice near the channel bottom, through the region of the channel designed to contain cell cultures: the “cell-containing channel region”), as well as the input oxygen conditions; (**b**) Simulated oxygen levels within the channel at 0 µL/min fluid flow. Average oxygen level in this region was 0.03%, while the maximum was 0.06%; (**c**) Simulated oxygen levels within the channel at 0.5 µL/min fluid flow. Average oxygen level in this region was 0.03%, while the maximum was 0.05%; (**d**) Simulated oxygen levels within the channel at 1 µL/min fluid flow. Average oxygen level in this region was 0.03%, while the maximum was 0.05%; (**e**) Simulated oxygen levels within the channel at 10 µL/min fluid flow. Average oxygen level in this region was 2.01%, while the maximum was 7.33%; (**f**) Simulated oxygen levels within the channel at 15 µL/min fluid flow. Average oxygen level in this region was 3.86%, while the maximum was 11.2%; (**g**) False-colour images of measured oxygen profiles near the chip inlet, at 0.5, 1, 10, and 15 µL/min fluid flow, overlaid upon a brightfield microscope image of the channel. Average oxygen levels in the sensor patch regions were 0.01%, 0.009%, 6.5%, and 9.9% at 0.5, 1, 10, and 15 µL/min fluid flow, respectively.

Finite-element modelling was used to validate the ability of the oxygen control channels to control the oxygen levels within the cell culture channel, independent of the environment outside of the chip and the oxygenation of the liquid supplied to the cell culture and hydration channels. In all simulations, the environment outside of the chip and the liquid entering the channels were taken to be equilibrated with 21% oxygen. Liquid flow rates of 0, 0.5, 1, 10, and 15 μL/min were simulated and compared, and the oxygen levels near the bottom of the cell culture channel, in the part of the channel designed to contain the cell cultures (the ‘cell-containing channel region’, after the entrance/equilibration length), were extracted. The average velocity in this same region of the chip but at the channel middle rather than channel bottom was also extracted. Experimental measurements using the device and integrated sensors were conducted to compare with the simulated results. Figure 5 presents the simulation geometry showing the region of the cell culture channel analyzed (the “cell-containing channel region”) Figure 5a, the results of these simulations Figure 5b–f, and the measurements Figure 5g.

The data extracted from these simulations is summarized in Table 1. The average velocity ū, and average (Ō_2_) and maximum (O_2 max_) oxygen levels in the cell-containing channel region were extracted from the simulations and plotted. The Reynolds number (Re) was calculated from ū (Re = ūD_h_ρ/μ, with D_h_ indicating the channel hydraulic diameter and ρ and μ denoting the density and viscosity of the fluid, respectively).

**Table 1:** Simulated average velocity ū in the channel middle, Reynolds number Re, Péclet number Pe, and the average (Ō_2_) and maximum (O_2 max_) oxygen level in the bottom of the cell-containing channel region for different volumetric liquid flow rates Q. All values determined from the results of the simulations presented in Figure 5.

Q (µL/min)	ū (m/s)	Re	Pe	Ō_2_ (%)	O_2 max_ (%)
0	0	0	0	3.46 × 10^−2^	5.75 × 10^−2^
0.5	1.10 × 10^−4^	2.97 × 10^−5^	0.150	3.41 × 10^−2^	5.10 × 10^−2^
1	2.19 × 10^−4^	5.94 × 10^−5^	0.301	3.46 × 10^−2^	4.90 × 10^−2^
3	6.57 × 10^−4^	1.78 × 10^−4^	0.903	8.96 × 10^−2^	0.503
5	1.10 × 10^−3^	2.97 × 10^−4^	1.50	0.382	2.12
10	2.19 × 10^−3^	5.94 × 10^−4^	3.01	2.01	7.33
15	3.29 × 10^−3^	8.91 × 10^−4^	4.51	3.86	11.2

A Péclet number Pe was also defined for the system and calculated from ū, representing the ratio between the diffusion time (1-D simplification) across the channel height (100 µm in the simulation geometry) and the convection time for the fluid to travel the length of the equilibration region at the beginning of the channel, before the cell-containing region begins (1.3 mm):

(4) Pe=h22DLequ¯=h2u¯2LeqD

where *h* is the channel height, *L_eq_* is the equilibration length at the start of the channel before the cell traps begin, and *D* is the diffusion coefficient of oxygen in water.The laminar Reynolds numbers remain much less than 1 for the extent of the flow rates tested. As the calculated Péclet numbers are for the cell culture channel only, and do not take into account the full system, the simulation results (Ō_2_, O_2 max_ as well as the plots of the oxygen levels in the cell-containing channel region shown in Figure 5b–f) better reflect the experimental system. The simulation results indicate that accurate control of the channel oxygen levels is achievable using the gas control channels for flow rates ≤1 µL/min (the operating regime of the device); these results are validated by the experimental findings. The transition point appears to be near 3 µL/min, where the maximum oxygen level in the cell-containing region is approximately 0.5%. After this point at higher flow rates (5, 10 and 15 µL/min), the faster liquid flow brings higher oxygen levels with it into the cell-containing channel region. These results also fit with our expectation of diffusion-based control over mass transport from the Péclet numbers calculated at flow rates ≤1 µL/min, given our entrance/equilibration length at the beginning of the cell culture channel. This control and isolation from the environment is essential for creating time-varying oxygen profiles with fast switching times, as if it is not achieved, the diffusion times through the PDMS bulk (~7 mm in thickness, leading to much longer time scales on the order of 1 h by a 1-D diffusion calculation) will begin to have an effect. Isolation from the input liquid oxygen level is also important, as it impacts the levels of hypoxia achievable using the device without pre-equilibrating the cell culture media perfused to the chip (which could also impact the equilibration times for the system).

### 3.4. Oxygen Gradients within the Microfluidic Device

**Figure 6:**
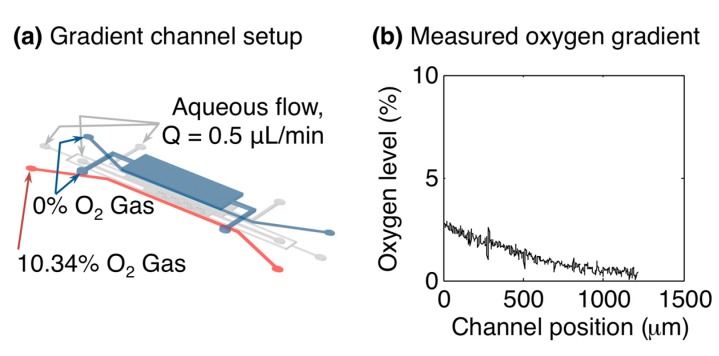
Oxygen gradient formation within the microfluidic oxygen control device. (**a**) Microfluidic channel setup for gradient measurement. L1GL was supplied with 10.34% oxygen, while L1GR and L3G were supplied with 0%; (**b**) Oxygen gradient measured inside the cell culture channel by the *in situ* sensors, after ~10 min equilibration. The plot shows the oxygen level *vs.* the position across the width of the cell culture channel (*i.e.*, in the direction between L1GL and L1GR).

The individually-addressable gas control channels within the microfluidic device offer the opportunity to create spatial oxygen gradients across the cell culture channel. An example oxygen gradient measurement within the microfluidic oxygen control device is presented in Figure 6. A stable gradient across the width of the cell culture channel is formed after equilibrating for approximately 10 min.

### 3.5. Oxygen Switching Times and Time-Varying Oxygen Profiles

The equilibration time within the gas control device (after switching the gas control inlets from 7.33% oxygen to 0% oxygen) was simulated to stabilize to less than 0.5% of the initial oxygen value within 530 s. Equilibration curves for starting oxygen levels (O_2 start_) of 7.33%, 10%, and 21% and flow rates (Q) of 0, 0.5, and 1 µL/min were simulated. Due to the dominant control of the channel oxygen by the control channels at the flow rates simulated, neither the O_2 start_ nor Q significantly affected the equilibration curves within the simulated range. This finding demonstrates the control over the oxygen levels achieved by the gas control channels and is consistent with the Péclet number calculation of Table 1 and simulation results of Figure 5. The oxygen level at time 530 s only varied by 0.3% of the starting value between all of the simulations, with the flow rate showing no effect on the curves, and the curves for O_2 start_ = 7.33% and 21% reaching 0.5% and 0.2% of O_2 start_, respectively at t = 530 s.

**Figure 7:**
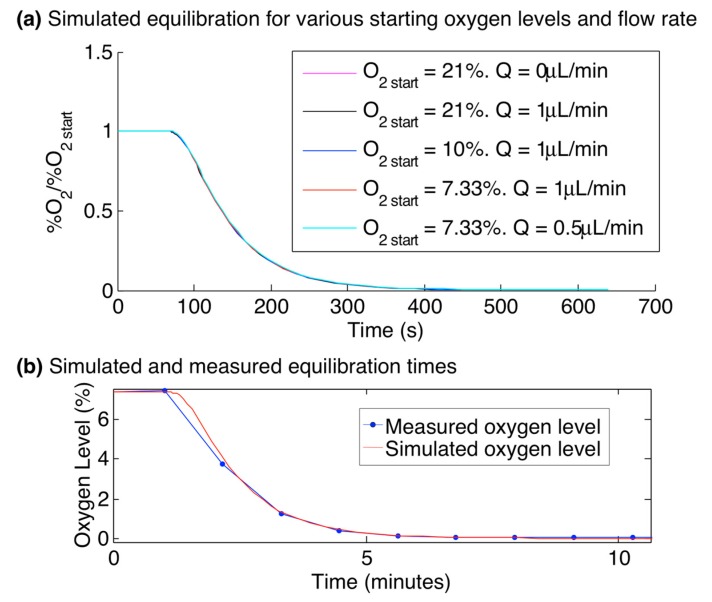
Simulated and experimental oxygen equilibration times within the multilayer microfluidic device. (**a**) Time-dependent simulation results, showing the oxygen level at the centre of the cell culture channel after the oxygen level supplied to the gas control channels L1GL, L1GR, and L3G was switched from O_2 start_ to 0% at time 60 s, for values of O_2 start_ ranging from 7.33% to 21%, and flow rates Q ranging from 0 to 1 µL/min. Neither the O_2 start_ nor Q significantly affected the equilibration curves (e.g., the oxygen level at time 530 s only varied by 0.3% of O_2 start_ between all of the simulations, with the flow rate showing no effect on the curves, and the curves for O_2 start_ = 7.33% and 21% reaching 0.5% and 0.2% of O_2 start_, respectively at t = 530 s); (**b**) Simulation and measurement of the oxygen levels within the oxygen control device, after the input gas level changed from 7.33% to 0% oxygen at time 1 min. System shows equilibration to less than 0.5% of the initial oxygen level within 530 s.

This simulated switching time was also verified experimentally by changing the gas level supplied to the gas control channels from 7.33% to 0% and monitoring the oxygen levels in the cell culture channel using the integrated sensors during the equilibration period. Figure 7 presents the simulated equilibration curves, normalized to O_2 start_, for three values of O_2 start_ and Q, as well as the oxygen levels measured by the integrated sensors during an oxygen switching experiment with a fluid flow rate of 0.5 µL/min overlaid upon the simulated values (also using O_2 start_ = 7.33% using Q = 0.5 µL/min), showing excellent agreement. We believe that the discrepancy between the measured and simulated curves just after the switch in gas level is due to the sampling rate used in the measurement (1 minute between imaging cycles, with a line used to connect the data points for visualization). The oxygen levels equilibrated to within 0.5% of the initial value after 530 s. As the equilibration time is very consistent between the experimental results and the model, we do not believe that the sensor response time has a significant impact on the measured equilibration.

**Figure 8:**
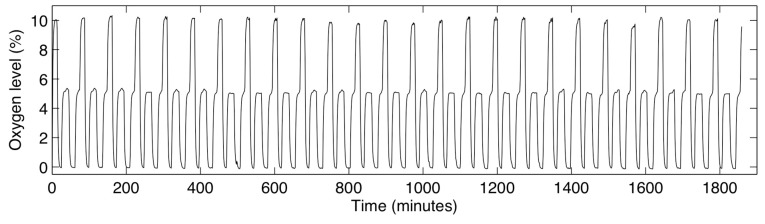
Measured oxygen levels within the cell culture channel during a cyclic, time-varying oxygen profile in the device, lasting 31 h.

The switching times of 530 s achievable with the multilayer device permit the formation of complex, cyclic oxygen profiles within the cell culture channel. An example measurement of this kind of profile is presented in Figure 8. For this measurement, a cyclic profile repeating the following oxygen levels was supplied to all three gas control channels: 10% oxygen was supplied for 800 s, followed by 0% for 700 s, 5% for 1200 s, 0% for 1000 s, and 5% for 750 s. This profile was repeated for 31 h and monitored with the integrated sensors in the cell culture channel by imaging every 60 s. Water was perfused into the cell culture and hydration channels at a rate of 0.5 µL/min throughout the course of the experiment using a syringe pump to simulate the perfusion of fresh media to cells in culture. Slight variations in the 5% and 10% readings are likely due to the mixing error of the gas system (which had a tolerance of 0.5%). The measured oxygen levels appear to be stable over the course of the experiment, demonstrating the suitability of the system for applications in long-term cell culture.

## 4. Conclusions/Outlook

We have demonstrated a microfluidic oxygen control device with integrated ratiometric oxygen sensors suitable for long-term cell culture applications. Long-term experiments with oxygen monitoring require good sensor stability over long periods of time with repeated measurements; we have demonstrated how sensor stability can be improved by integrating ratiometric sensors, as well as by pre-photobleaching the sensors and by using a microscope shutter to limit excitation exposure of the dye. With both finite-element modelling and experimental data we have shown that the device permits excellent control of the oxygen levels in a chamber designed for cell culture, independent of the oxygenation of the environment outside the chip. We studied the effects of oxygenated media flow rates on the simulated and measured oxygen profiles, and found an operating regime for the device (flow rates of 0.5–1 µL/min) wherein the oxygen levels in the cell culture channel after the entrance length are independent of flow rate or oxygenation of the flowing media. The device showed an oxygen equilibration time of less than 10 min (showing excellent agreement between modeled and measured equilibration times), permitting the creation of complex time-varying oxygen profiles with switching times on biologically-relevant timescales of a few cycles/hour.

Future work will involve applying the device to biological systems to recreate similar oxygen profiles to those that have been measured in tumours, studying their effects on tumour cells *in vitro*. Alginate beads containing 3-D cell cultures will be immobilized in the trapping structures on-chip and exposed to various spatial and temporally-varying oxygen profiles. Future work could also involve modifying the microfluidic device as necessary to accommodate more cell cultures, or optimizing the microfluidic design (chamber and membrane dimensions) or sensor composition (dye and matrix) for specific applications such as faster switching or severely hypoxic environments.
